# Supplementary material for: Selective activation of Gαob by an adenosine A1 receptor agonist elicits analgesia without cardiorespiratory depression
Source: Nat Commun. 2022 Jul 18;13:4150. doi: 10.1038/s41467-022-31652-2 (PMC9293909; doi:10.1038/s41467-022-31652-2)
Supplement: Supplementary file 1 — Supplementary Information [file 41467_2022_31652_MOESM1_ESM.pdf]

# Supplementary Information for:

# Selective activation of Gαob by an adenosine $A_1$ receptor agonist elicits analgesia without cardiorespiratory depression

Mark J. Wall[1*^], Emily Hill[1^], Robert Huckstepp[1^], Kerry Barkan[2^], Giuseppe Deganutti[3,4^], Michele Leuenberger[5^], Barbara Preti[5^], Ian Winfield[2^], Sabrina Carvalho[2], Anna Suchankova[2], Haifeng Wei[6], Dewi Safitri[2,7], Xianglin Huang[2], Wendy Imlach[8], Circe La Mache[1], Eve Dean[1], Cherise Hume[1], Stephanie Hayward[1], Jess Oliver[1], Fei-Yue Zhao[6], David Spanswick[6,8,9], Christopher A. Reynolds[3,4], Martin Lochner[5], Graham Ladds[2*] and Bruno G. Frenguelli[1*]

*Corresponding authors

^These authors contributed equally to this study

Correspondence should be addressed to:

Mark J. Wall mark.wall@warwick.ac.uk

Graham Ladds grl30@cam.ac.uk

Bruno G. Frenguelli b.g.frenguelli@warwick.ac.uk

Supplementary Figures and legends 1 – 11

Supplementary References 1 – 3

**Supplementary Table 1. Binding affinities and efficacies at human $A_1R$, $A_{2A}R$ and $A_3R$ expressed in CHO-KI cells**

| | hA1R | | | hA2AR | | | hA3R | | |
|---|---|---|---|---|---|---|---|---|---|
| | pKi[a] | pIC50[b] | Range[c] | pKi[d] | pEC50[b] | Range[c] | pKi[d] | pIC50[b] | Range[c] |
| **Adenosine** | **5.02 ± 0.10**<br>- | **8.45 ± 0.2**<br>- | **55.7 ± 4.0**<br>- | **5.74 ± 0.11**<br>- | **6.09 ± 1.3**<br>- | **38.1 ± 3.16**<br>- | **6.04 ± 0.1**<br>- | **8.34 ± 0.2**<br>- | **39.8 ± 2.3**<br>- |
| **CPA** | **6.65 ± 0.14*****<br>*P < 0.001* | **9.26 ± 0.3*****<br>*P < 0.001* | **48.97 ± 0.7**<br>- | **N.D**<br>- | **N.D**<br>- | **N.D**<br>- | **<4.0#**<br>- | **N.R.**<br>- | **N.R.**<br>- |
| **NECA** | **6.45 ± 0.06****<br>*P = 0.0075* | **9.05 ± 0.2*****<br>*P < 0.001* | **34.33 ± 4.0**<br>- | **6.36 ± 0.09**<br>- | **7.94 ± 0.1****<br>*P = 0.032* | **42.99 ± 4.32**<br>- | **6.82 ± 0.12**<br>- | **9.2 ± 0.31****<br>*p = 0.81* | **33.43 ± 3.1**<br>- |
| **HOCPA** | **5.81 ± 0.16****<br>*P = 0.0098* | **9.08 ± 0.1****<br>*P = 0.0045* | **60.52 ± 1.5**<br>- | **<4.0#**<br>- | **5.49 ± 0.1***<br>*P = 0.46* | **41.66 ± 6.1**<br>- | **6.23 ± 0.2**<br>- | **7.61 ± 0.22*****<br>*P = 0.31* | **45.0 ± 2.1**<br>- |
| **BnOCPA** | **6.47 ± 0.11*****<br>*P < 0.001* | **9.17 ± 0.3*****<br>*P < 0.001* | **49.0 ± 0.66**<br>- | **<4.0#**<br>- | **5.60 ± 0.01**<br>- | **49.0 ± 4.6**<br>- | **<4.0#**<br>- | **N.R.**<br>- | **N.R.**<br>- |

Average data ± SEM of 3 - 19 individual replicates

[a] Negative logarithm of agonist concentration displacing 50% bound [$^3$H]-DPCPX

[b] Negative logarithm of agonist concentration producing half-maximal response

[c] Range of response observed upon agonist stimulation, as a percentage of response obtained upon stimulation with 10 μM forskolin

[d] Negative logarithm of agonist concentration displacing 50% bound

[#] Full estimates of the binding constant were not possible due to failure to generate a full inhibition curve.

N.D. – Not determined, N.R. – No response.

Statistical difference between each agonist and adenosine was calculated using a one-way ANOVA with Dunnett's post-test (** P < 0.01; *** P < 0.001).

**Supplementary Table 2. Binding affinities and efficacies at rat $A_1R$, $A_{2A}R$ and $A_3R$ expressed in CHO-KI cells**

| | rA1R | | | rA2AR | | | rA3R | | |
|---|---|---|---|---|---|---|---|---|---|
| | pKi[a] | pIC50[b] | Range[c] | pKi[a] | pEC50[b] | Range[c] | pKi[d] | pIC50[b] | Range[c] |
| **Adenosine** | **5.41 ± 0.18**<br>- | **7.63 ± 0.1**<br>- | **34.40 ± 1.04**<br>- | **5.74 ± 0.11**<br>- | **7.58 ± 0.18**<br>- | **37.78 ± 2.76**<br>- | **5.89 ± 0.09**<br>- | **7.17 ± 0.18**<br>- | **66.05 ± 5.4**<br>- |
| **CPA** | **6.80 ± 0.14*****<br>*P < 0.001* | **9.47 ± 0.16*****<br>*P < 0.001* | **36.31 ± 2.57**<br>- | **<4.0#**<br>- | **5.55 ± 0.19*****<br>*P < 0.001* | **39.45 ± 3.47**<br>- | **N.D**<br>- | **7.41 ± 0.13**<br>- | **68.79 ± 3.67**<br>- |
| **NECA** | **6.32 ± 0.13****<br>*P = 0.0087* | **8.65 ± 0.77****<br>*P < 0.00* | **37.17 ± 5.49**<br>- | **6.36 ± 0.09**<br>- | **8.37 ± 0.18***<br>*P = 0.012* | **37.78 ± 2.65**<br>- | **6.43 ± 0.11***<br>*P = 0.041* | **8.81 ± 0.18****<br>*P = 0.0017* | **63.02 ± 5.14**<br>- |
| **HOCPA** | **6.27 ± 0.14*****<br>*P < 0.001* | **9.01 ± 0.01*****<br>*P < 0.001* | **34.40 ± 1.59**<br>- | **4.86 ± 0.12****<br>*P = 0.0034* | **5.69 ± 0.20*****<br>*P < 0.001* | **48.61 ± 6.42***<br>*P = 0.044* | **6.17 ± 0.02***<br>*P = 0.022* | **7.41 ± 0.12**<br>- | **68.79 ± 3.70**<br>- |
| **BnOCPA** | **6.25 ± 0.16****<br>*P = 0.0031* | **8.92 ± 0.14*****<br>*P < 0.001* | **39.45 ± 1.11**<br>- | **5.03 ± 0.11****<br>*P < 0.0084* | **5.00 ± 0.13*****<br>*P < 0.001* | **37.15 ± 2.76**<br>- | **5.27 ± 0.12**<br>- | **6.73 ± 0.13***<br>- | **65.16 ± 3.80**<br>- |

Average data ± SEM of 3 - 19 individual replicates

[a] Negative logarithm of agonist concentration displacing 50% bound CA200645

[b] Negative logarithm of agonist concentration producing half-maximal response

[c] Range of response observed upon agonist stimulation, as a percentage of response obtained upon stimulation with 10 μM forskolin

[d] Negative logarithm of agonist concentration displacing 50% bound AV039

[#] Full estimates of the binding constant were not possible due to failure to generate a full inhibition curve.

N.D. – Not determined.

Statistical difference between each agonist and adenosine was calculated using a one-way ANOVA with Dunnett's post-test (* P < 0.05; ** P < 0.01; *** P < 0.001).

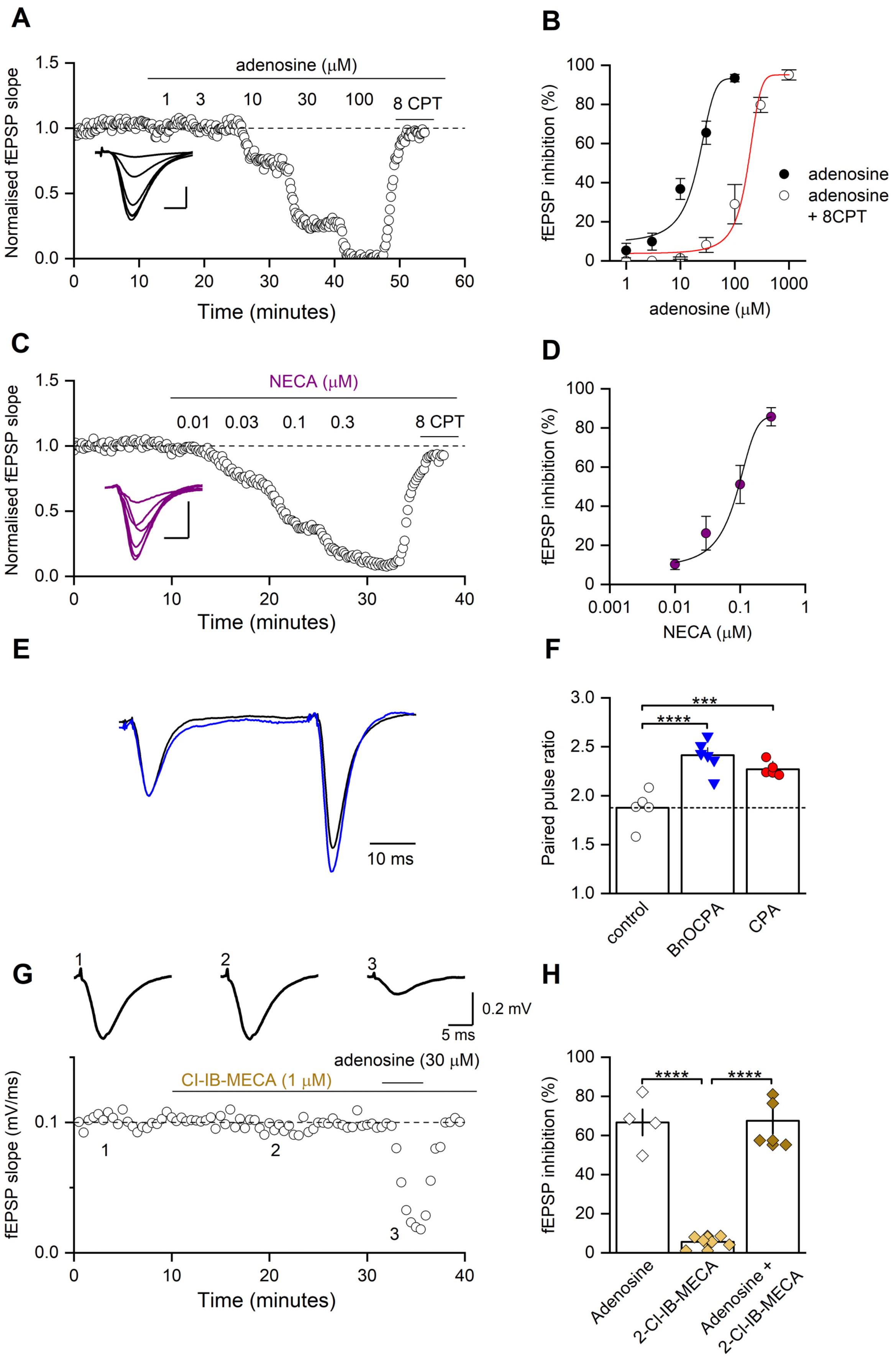


**Supplementary Fig. 1. $A_1R$, but not $A_3R$, agonists inhibit excitatory synaptic transmission at hippocampal synapses.**

**Supplementary Fig. 1. $A_1R$, but not $A_3R$, agonists inhibit excitatory synaptic transmission at hippocampal synapses.**

**A**, Increasing concentrations of adenosine reduced fEPSP slope, an effect reversed by the $A_1R$ antagonist 8CPT (2 µM). Inset, superimposed fEPSP averages in control and in increasing concentrations of adenosine. Scale bar measures 5 ms and 0.25 mV. **B**, Concentration-response curve for adenosine ($IC_{50}$ = 20 ± 4.3 µM, *n* =11 slices) and for adenosine with 2 µM 8CPT ($IC_{50}$ = 125 ± 10 µM *n* = 5 slices). **C**, Increasing concentrations of the $A_1R$ agonist NECA reduced fEPSP slope, an effect reversed by 8CPT (2 µM). Inset, superimposed fEPSP averages in control and in increasing concentrations of NECA. Scale bar measures 5 ms and 0.25 mV. **D**, Concentration-response curve for NECA ($IC_{50}$ = 8.3 ± 3 nM, *n* =11 slices). **E**, Example of average (5 traces) superimposed paired-pulse fEPSP waveforms (50 ms inter-pulse interval) in control (black trace) and in the presence of BnOCPA (100 nM; blue trace). The fEPSP waveforms have been normalised to the amplitude of the first fEPSP in control. BnOCPA increased paired-pulse facilitation, indicative of a BnOCPA-induced reduction in the probability of glutamate release. **F**, Data summary. For a paired-pulse interval of 50 ms, the paired-pulse ratio was significantly increased (one-way ANOVA; $F(2, 14) = 21.72$; $P = 5.11 \times 10^{-5}$) from 1.88 ± 0.07 in control (*n* = 6 slices) to 2.41 ± 0.07 in BnOCPA (*n* = 6 slices, $P = 5.17 \times 10^{-5}$) and 2.27 ± 0.03 in CPA (60 nM; *n* = 5, P = 0.001). **G**, The potent and selective $A_3R$ agonist 2-Cl-IB-MECA had no effect on the fEPSP even at a high concentration (1 µM) and did not prevent adenosine (30 µM) from inhibiting synaptic transmission to an extent comparable to that seen in the absence of 2-Cl-IB-MECA (Panels A, B). Data presented shows the time course of an exemplar experiment with the inset fEPSPs taken at the times indicated (1) before, (2) during 2-Cl-IB-MECA, and (3) during adenosine application in the continued presence of the selective $A_3R$ agonist. **H**, Summary for the effects of 2-Cl-IB-MECA (1 µM) on fEPSPs and on the depression caused by adenosine (*n* = 4 – 6 slices; one-way ANOVA; $F(2, 11) = 65.60$; $P = 7.71 \times 10^{-7}$). Adenosine (30 µM) and adenosine (30 µM) in the presence of 2-Cl-IB-MECA (1 µM) depressed the fEPSP to comparable levels (66.7 ± 6.7 % and 67.5 ± 6.5 %; P = 1), and to a significantly greater extent than that caused by 2-Cl-IB-MECA (5.6 ±1.1 %; $P = 3.63 \times 10^{-6}$ vs adenosine, and $P = 3.17 \times 10^{-6}$ vs adenosine plus 2-Cl-IB MECA. Averaged data is presented as mean ± SEM. ***, P < 0.001; ****, P < 0.0001.

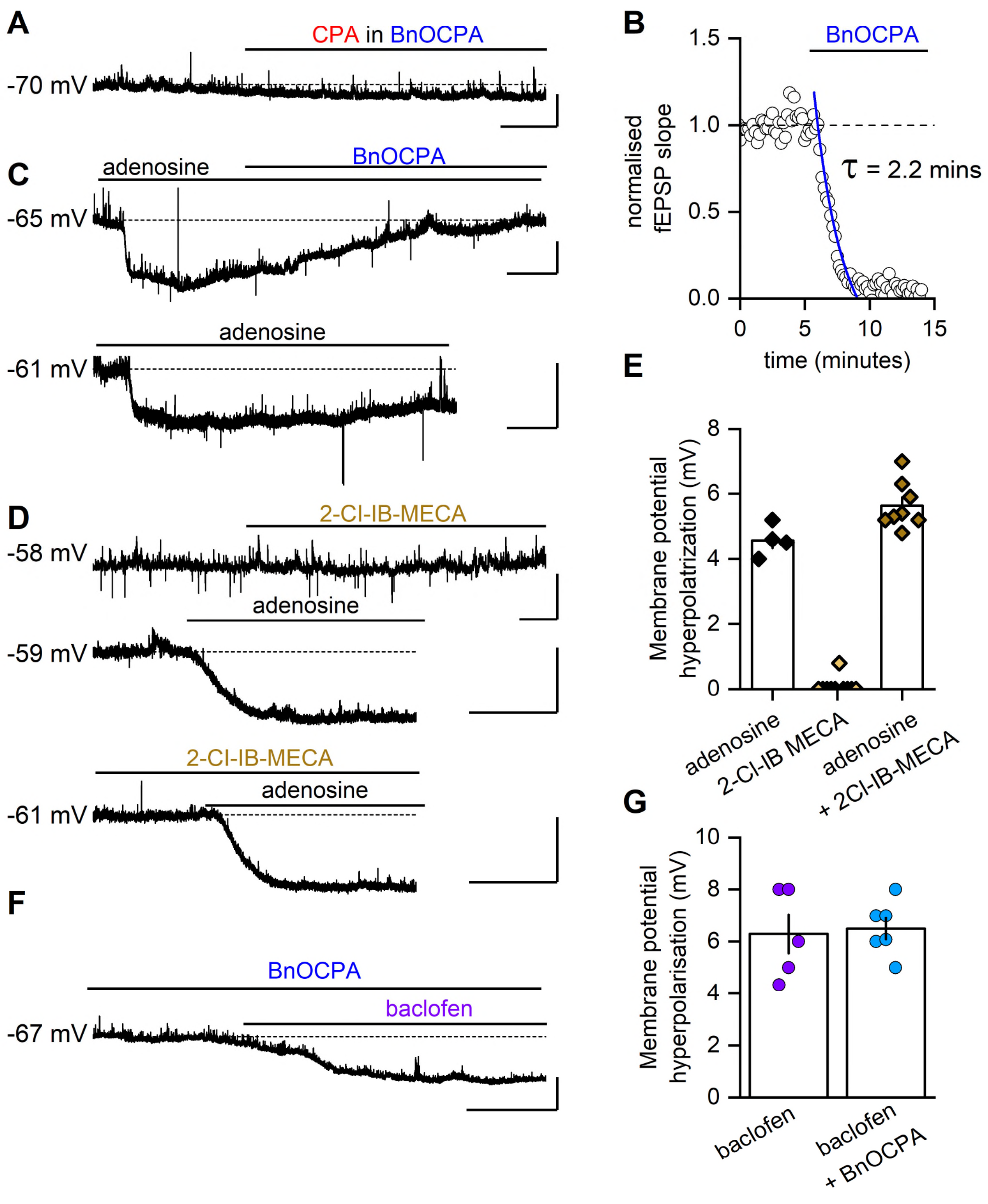


**Supplementary Fig. 2. BnOCPA, but not the A$_3$R agonist 2-Cl-IB-MECA, selectively inhibits membrane hyperpolarisation induced by prototypical A$_1$R agonists.**

**A**, Membrane potential trace recorded from a CA1 pyramidal cell. BnOCPA (300 nM) reduced the effect of CPA (300 nM; quantified in main text Fig. 1i). **B,** The same solution of BnOCPA (300 nM), which had no effect on membrane potential, abolished synaptic transmission in a sister slice (inhibition fitted with a single exponential; $\tau$ = 2.2 mins). **C**, BnOCPA reversed the hyperpolarising effect of adenosine (100 μM; similar observations were made in 3 other cells), which (lower trace) cannot be accounted for by fatigue of adenosine-mediated hyperpolarisation (similar observations of sustained hyperpolarisations to adenosine were made in 2 other cells). **D**, 2-Cl-IB-MECA had no effect on membrane potential even when applied at a high concentration (1 μM). Moreover, the membrane

hyperpolarisation caused by adenosine (30 μM) was not affected by prior incubation and co-application with 2-Cl-IB-MECA (1 μM). **E**, Summary of data from 9 – 11 cells showing that 2-Cl-IB-MECA does not affect membrane potential, nor does it prevent adenosine from inducing membrane hyperpolarisation, in contrast to BnOCPA, which reverses adenosine-mediated membrane hyperpolarisation. Bonferroni post-hoc comparisons after a one-way ANOVA ($F(2,26) = 183.83$, $P = 4.441 \times 10^{-16}$) showed no significant (ns) difference between adenosine application in the absence or presence of 2-Cl-IB-MECA ($P = 0.621$), but significantly smaller hyperpolarisations to 2-Cl-IB-MECA compared to adenosine alone ($P = 3.029 \times 10^{-14}$), or adenosine in the presence of 2-Cl-IB-MECA ($P = 4.177 \times 10^{-15}$). ****; $P < 0.0001$. **F,** Application of baclofen (10 μM) in the presence of BnOCPA (300 nM) hyperpolarised the membrane potential (from -67 to -74 mV). Scale bars measure 5 mV and 25 s (2-Cl-IB-MECA) 50 s (CPA), 200 s (adenosine) or 100 s (baclofen). **G**, Data summary of baclofen/BnOCPA experiments. The mean hyperpolarisation produced by baclofen in the presence of BnOCPA was not significantly different (ns; two-tailed unpaired t-test) from that produced by baclofen in control conditions (6.5 ± 0.43 mV vs 6.3 ± 0.76 mV, $P = 0.774$, $n$ = 5 - 6 cells for each condition). Bar chart displays individual data points and mean ± SEM.

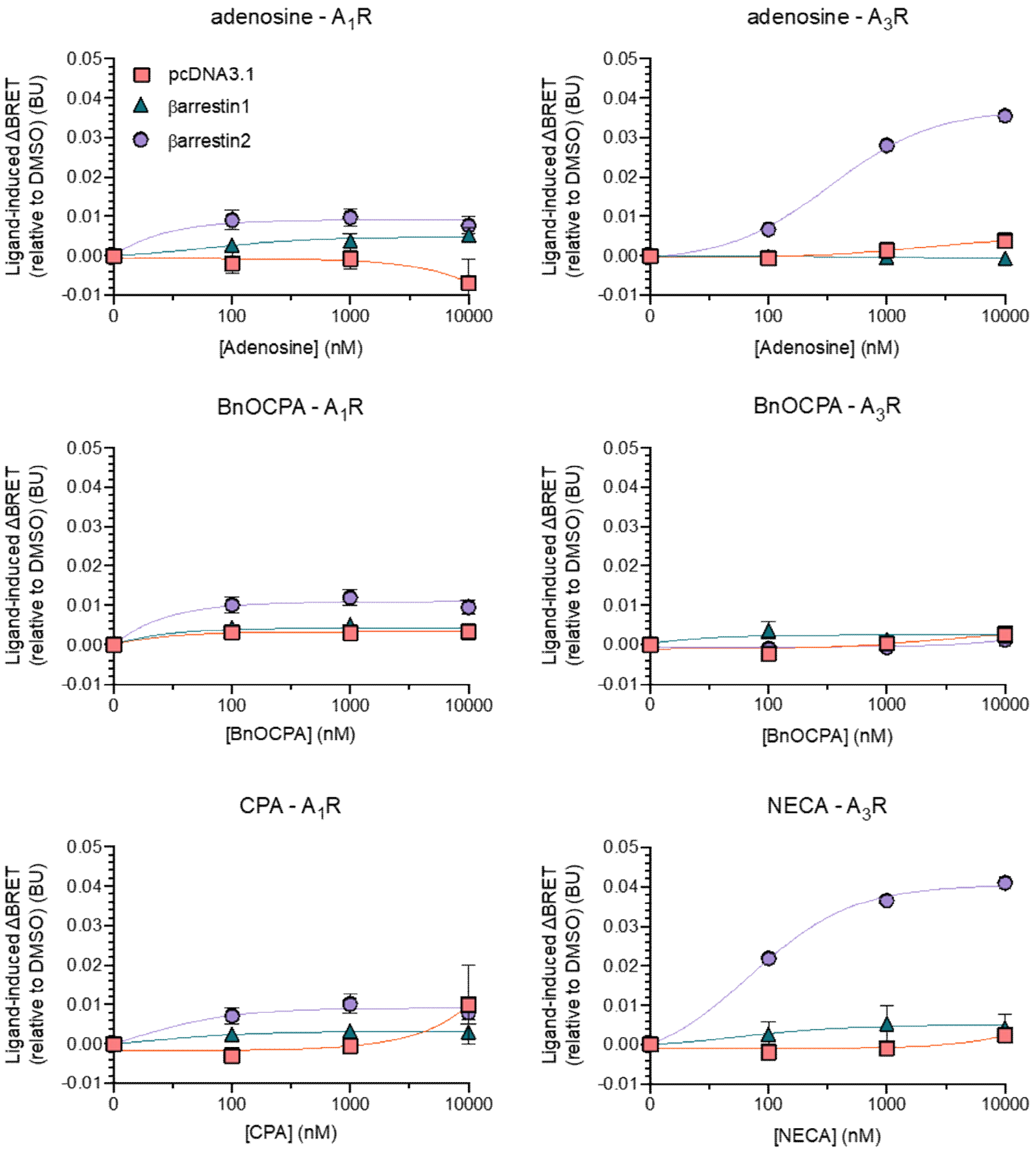


**Supplementary Fig. 3. β-arrestin1 or β-arrestin2 recruitment to the human $A_1R$ or $A_3R$.**

Interactions were detected via BRET using a C-terminally Nluc-tagged GPCR ($A_1R$, left panels, or $A_3R$, right panels) and C-terminally YFP-tagged β-arrestin1 or β-arrestin2, or pcDNA3.1 (negative control). Cells were stimulated with 3 agonists (top panels – adenosine; middle panels – BnOCPA; lower left – CPA; lower right – NECA). Note lack of either β-arrestin1 or β-arrestin2 recruitment to the $A_1R$ either by adenosine, CPA or BnOCPA, which yield BRET signals comparable to the vector control experiments (pcDNA3.1; top panels). $A_3R$ recruitment of β-arrestin2 is provided as a positive control for the BRET assay. Data are presented as mean values ± SEM from 4 individual replicates in each condition.

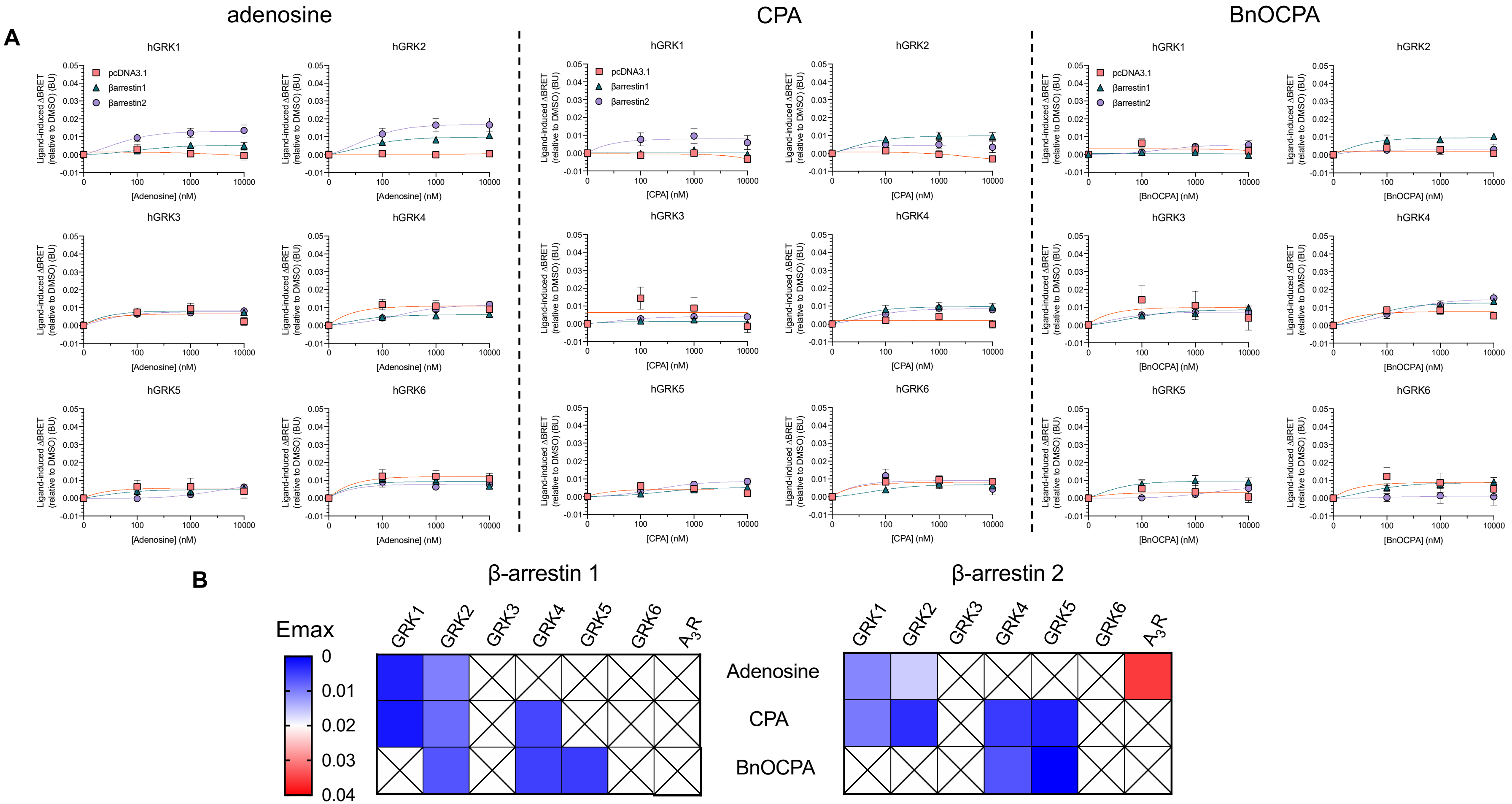


**Supplementary Fig. 4. GRK dependence of β-arrestin1 or β-arrestin2 recruitment to the $hA_1R$.**

**A**, Human G protein receptor kinase (hGRK) isoforms 1 – 6 were over expressed (5-fold relative to $A_1R$-Nluc), in the presence of control vector (pcDNA3.1) or β-arrestin1-YFP or β-arrestin2-YFP. BRET coupling was examined for each of these combinations for adenosine (left panels); BnOCPA (middle panels) or CPA (right panels). Data are presented as mean values ± SEM from 4-6 individual replicates in each condition. **B**, Heat map describing the peak maximum β-arrestin1 (left panel) and β-arrestin2 (right panel) recruitment for $hA_1R$ in the presence of the 6 GRK isoforms. $A_3R$ β-arrestin recruitment is included as a control. In all cases minimal β-arrestin recruitment was observed for the three agonists at the $A_1R$.

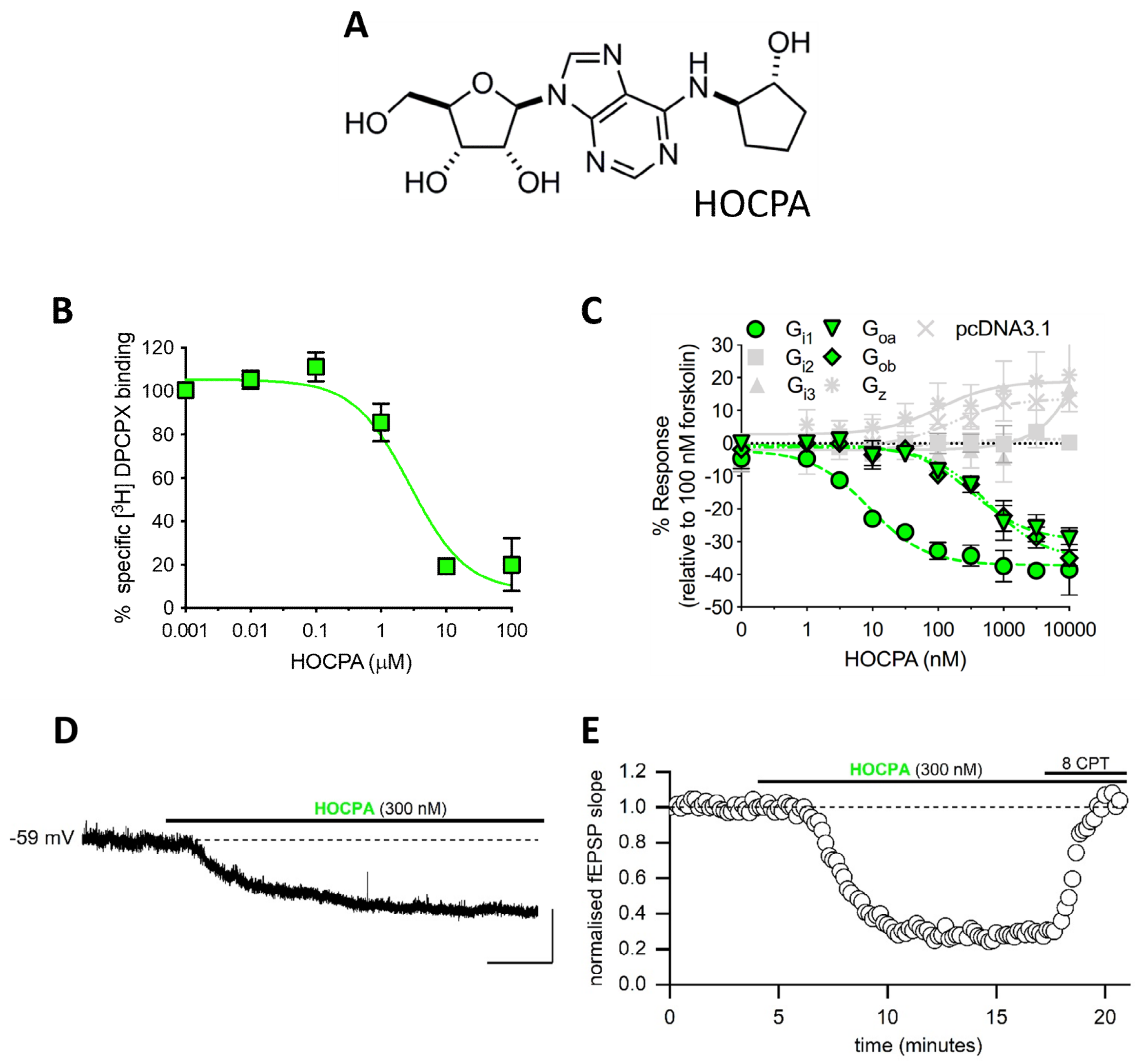


**Supplementary Fig. 5. HOCPA does not show Gα selectivity and does not discriminate between pre- and postsynaptic $A_1$Rs.**

**A**, Chemical structure of HOCPA. **B**, Binding of HOCPA was measured via its ability to displace [$^3$H]DPCPX from CHO-K1-h$A_1$R cells membranes. **C**, The ability of HOCPA to inhibit forskolin-stimulated (100 nM) cAMP production in PTX pre-treated (200 ng/ml) CHO-K1-h$A_1$R cells, transfected with PTX-insensitive Gi1, Gi2, Gi3, Goa, Gob, Gz or control (pcDNA3.1). In contrast to BnOCPA, HOCPA shows no selectivity between Goa and Gob. Data in **B** and **C** are presented as mean ± SEM of 4 individual replicates. **D**, Example membrane potential trace. HOCPA (300 nM) induced hyperpolarisation (the mean hyperpolarisation in 6 individual cells measured 5.3 ± 0.5 mV). Scale bars measure 5 mV and 50 s. **E**, Graph plotting normalised fEPSP slope against time for a single experiment. HOCPA caused a ~80 % reduction in fEPSP slope, which was reversed by the $A_1$R antagonist 8CPT (4 μM). Similar observations were made in 4 individual slices.

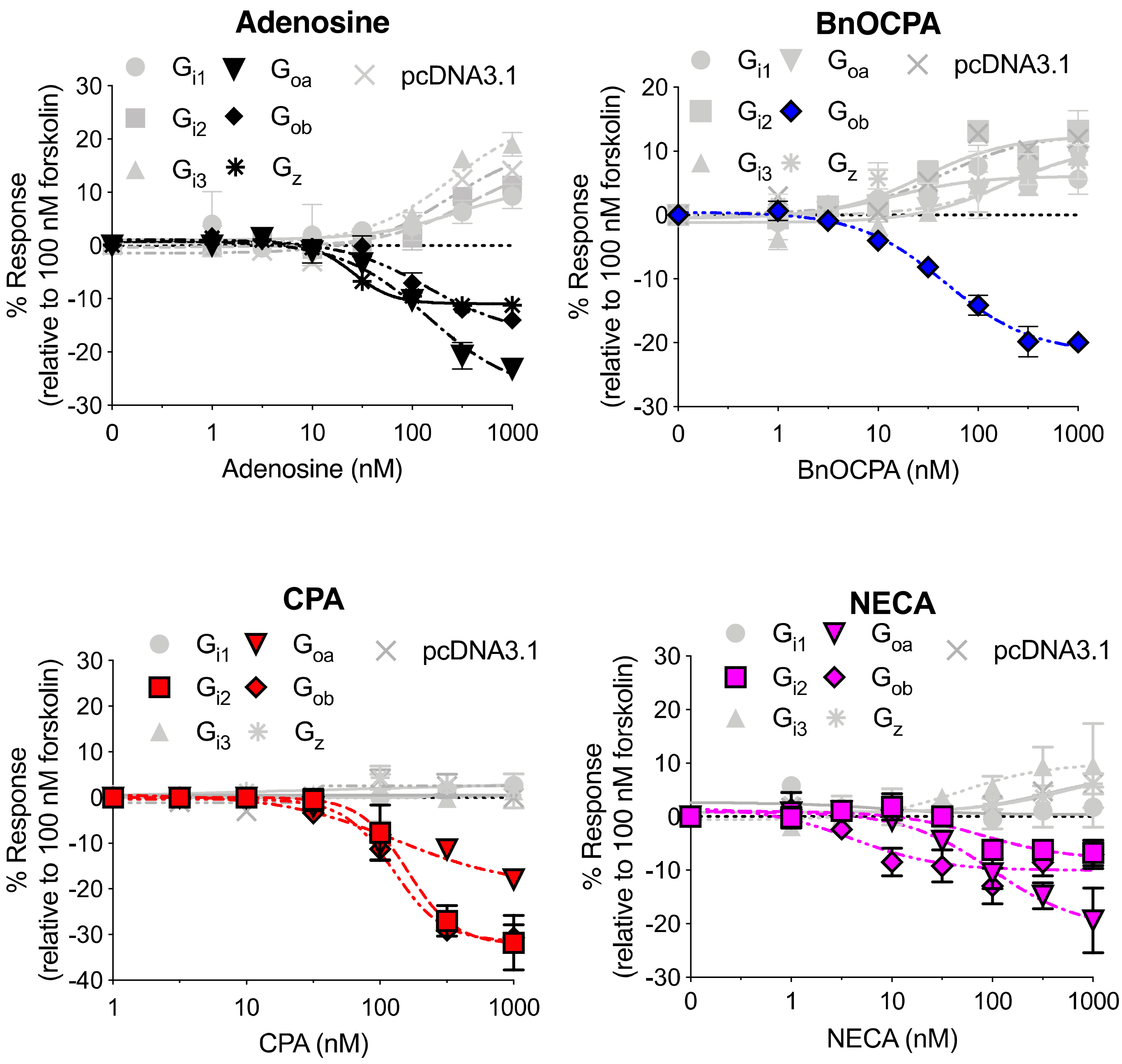


**Supplementary Fig. 6. Prototypical and atypical A$_1$R agonists display differing Gαi/o activation profiles.**

The ability of adenosine, BnOCPA, CPA and NECA to activate each individual Gi/o/z subtype was determined in CHO-K1-hA$_1$R cells, transfected with PTX-insensitive G proteins or control (pcDNA3.1). cAMP levels were measured following 30 minute co-stimulation with 100 nM forskolin and each agonist. Adenosine displayed an ability to inhibit cAMP production via activation of Gi2, Goa, Gob, and Gz; CPA and NECA via Gi2, Goa and Gob, and BnOCPA exclusively via Gob. Data represented as the average level of cAMP production relative to that observed upon stimulation with 100 nM forskolin, ± SEM, of *n* = 4 - 6 individual replicates. Stimulation of cAMP production reflects activation of endogenous Gs by the A$_1$R and is in agreement with previous observations[1-3].

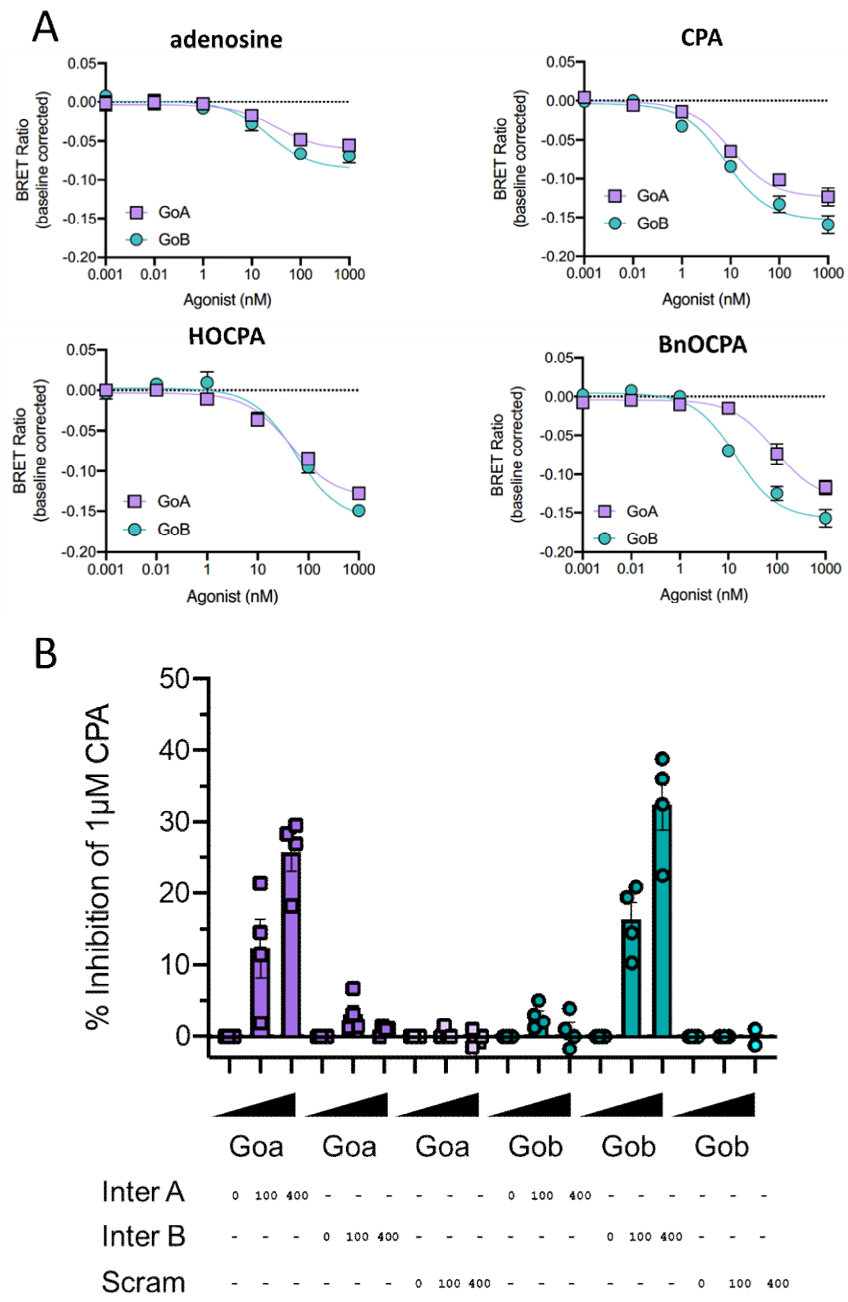


**Supplementary Fig. 7 TRUPATH assays of Goa and Gob activation and the influence of interfering peptides against Goa and Gob.**

**A** Concentration-response curves (from 6 - 8 biological replicates performed in duplicate) for the agonist-induced dissociation of Gα and Gβγ subunits in the TRUPATH BRET assay for Goa and Gob activation. Ratios have been baseline corrected with respect to a blank sample. Data are presented as mean values ± SEM. **B** Effects of increasing doses (in ng of plasmid) of interfering and scrambled peptides on the BRET ratio obtained from Goa and Gob in response to 1 μM CPA (4 biological replicates performed in duplicate). Inhibition of the CPA-induced BRET signal is only seen when the interfering peptide is used against its cognate Go isoform. The scrambled Goa peptide has no effect on the CPA-induced BRET signal induced by either Goa or Gob. Individual data points are presented, with the bar chart representing mean values ± SEM.

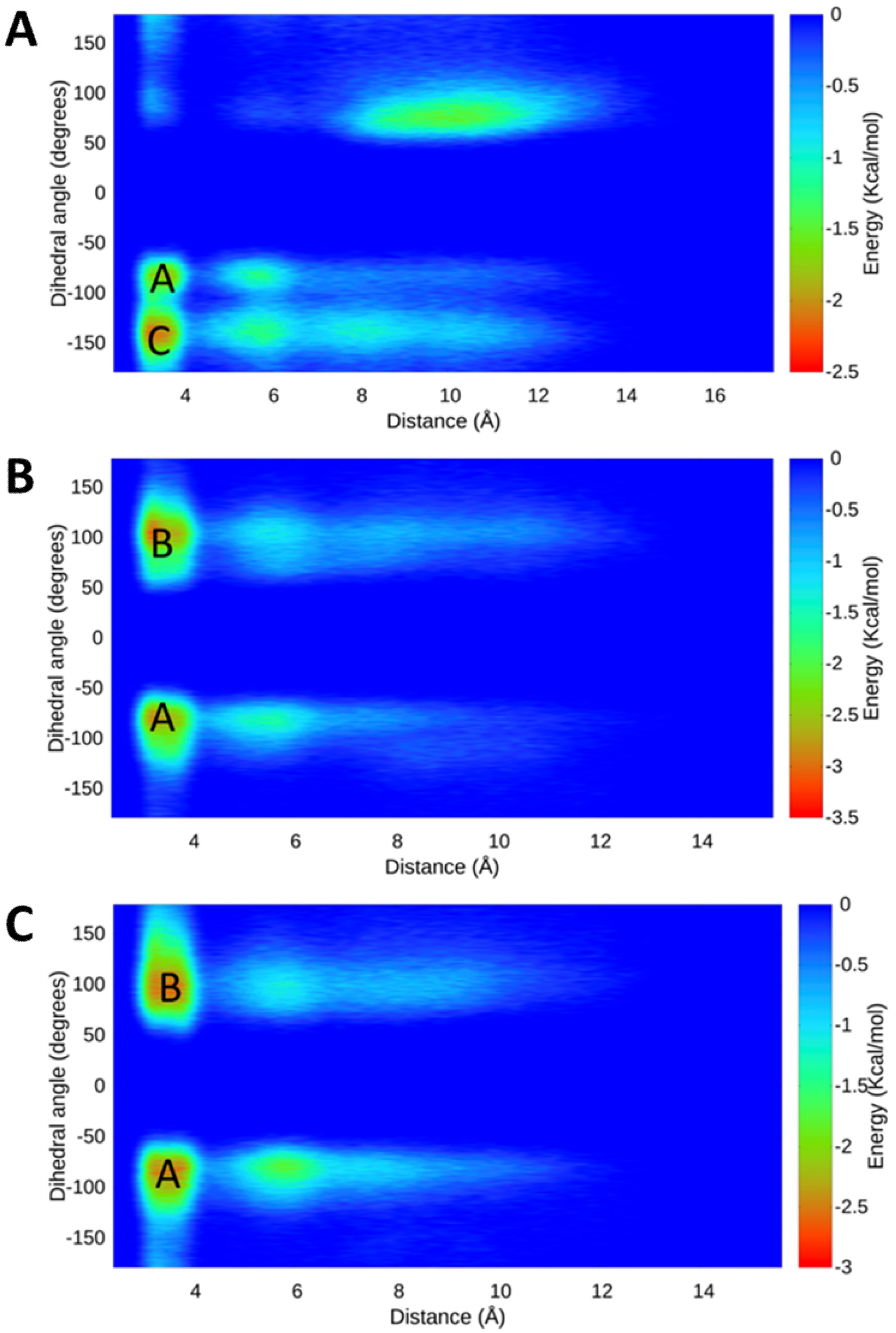


**Supplementary Fig. 8. Energy surfaces obtained from metadynamics simulations of BnOCPA**.

Energy surface obtained by integrating the Gaussian terms deposited during three well-tempered metadynamics replicas (panels **A**, **B** and **C**). X axes report the distance between the E172$^{ECL2}$ carboxyl carbon and the positively charged K265$^{ECL3}$ nitrogen atom; Y axes indicate the dihedral angle formed by the 4 atoms linking BnOCPA cyclopentyl ring to the phenyl moiety. The three energy minima (A, B and C) correspond to the three binding modes proposed for BnOCPA (Modes A, B, C in Fig. 3d to f, respectively).

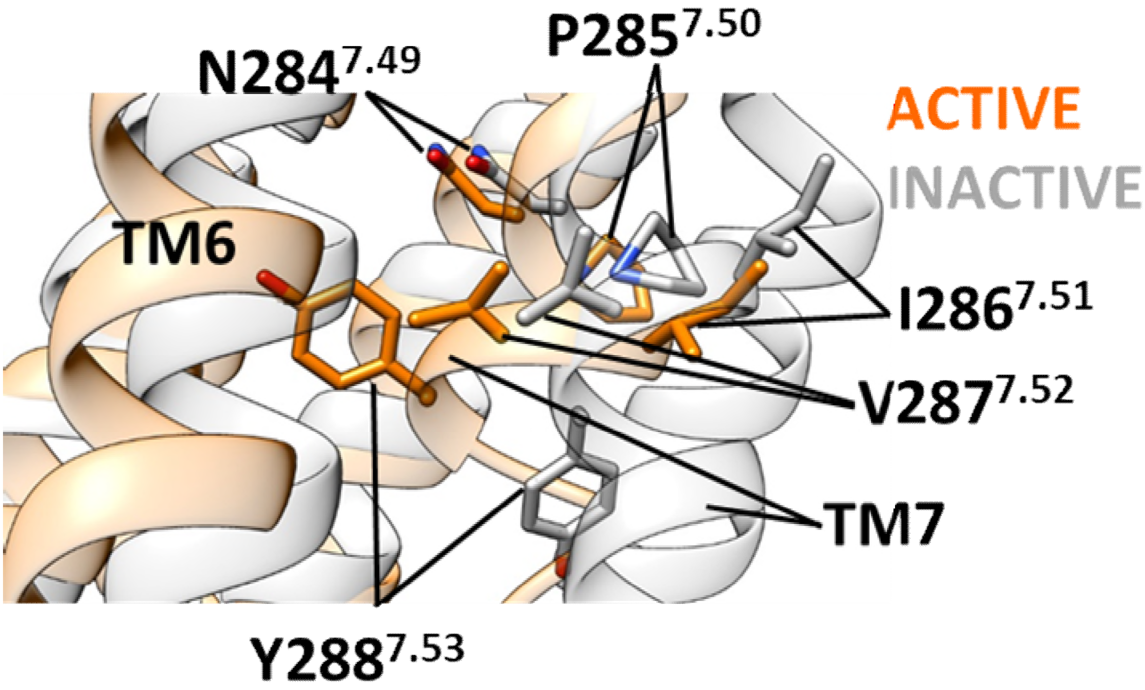


**Supplementary Fig. 9. The conserved NPXXY motif ($N^{7.49}$ PIV $Y^{7.53}$) in the $A_1R$**.
The root mean square deviation (RMSD) was computed with respect to the $A_1R$ inactive conformation. Compared to the inactive conformation (grey), in the active state (orange) the distal portion of TM7 is moved towards the TM bundle core (which is responsible for G protein binding). Starting from the active conformation (orange) and in absence of bound G protein, simulations should allow the structure to partially relax towards the inactive state (grey) with a dynamic influenced by the orthosteric ligand.

**Supplementary Table 3.** Transient hydrogen bonds between α4-β6 loop residue 317 (N317 in Goa, H317 in Gob), the α3-β5 loop residue D263, and the residue on H8 of the $A_1R$ (Ballesteros Weinstein enumeration in superscript).

| **$A_1R$ - Gα Interactions** | | | | | |
|---|---|---|---|---|---|
| | **Coupling Systems** | | | **Non-coupling Systems** | |
| | **Occupancy (%frames)** | | | **Occupancy (%frames)** | |
| **$A_1R$ - Gα hydrogen bond** | **BnOCPA mode D:Gob** | **BnOCPA mode B:Gob** | **HOCPA:Gob** | **BnOCPA mode D:Goa** | **BnOCPA mode B:Goa** |
| **H317-Q293$^{8.48}$** | **1.7** | **0.5** | **2.8** | **6.9** | **10.3** |
| **D263-Q293$^{8.48}$** | **0.4** | **0.4** | **1.5** | **9.2** | **0.6** |
| **K294$^{8.49}$-D263** | **0.0** | **0.0** | **0.1** | **4.0** | **2.9** |
| **R296$^{8.51}$-D263** | **0.1** | **0.5** | **0.0** | **10.7** | **0.0** |

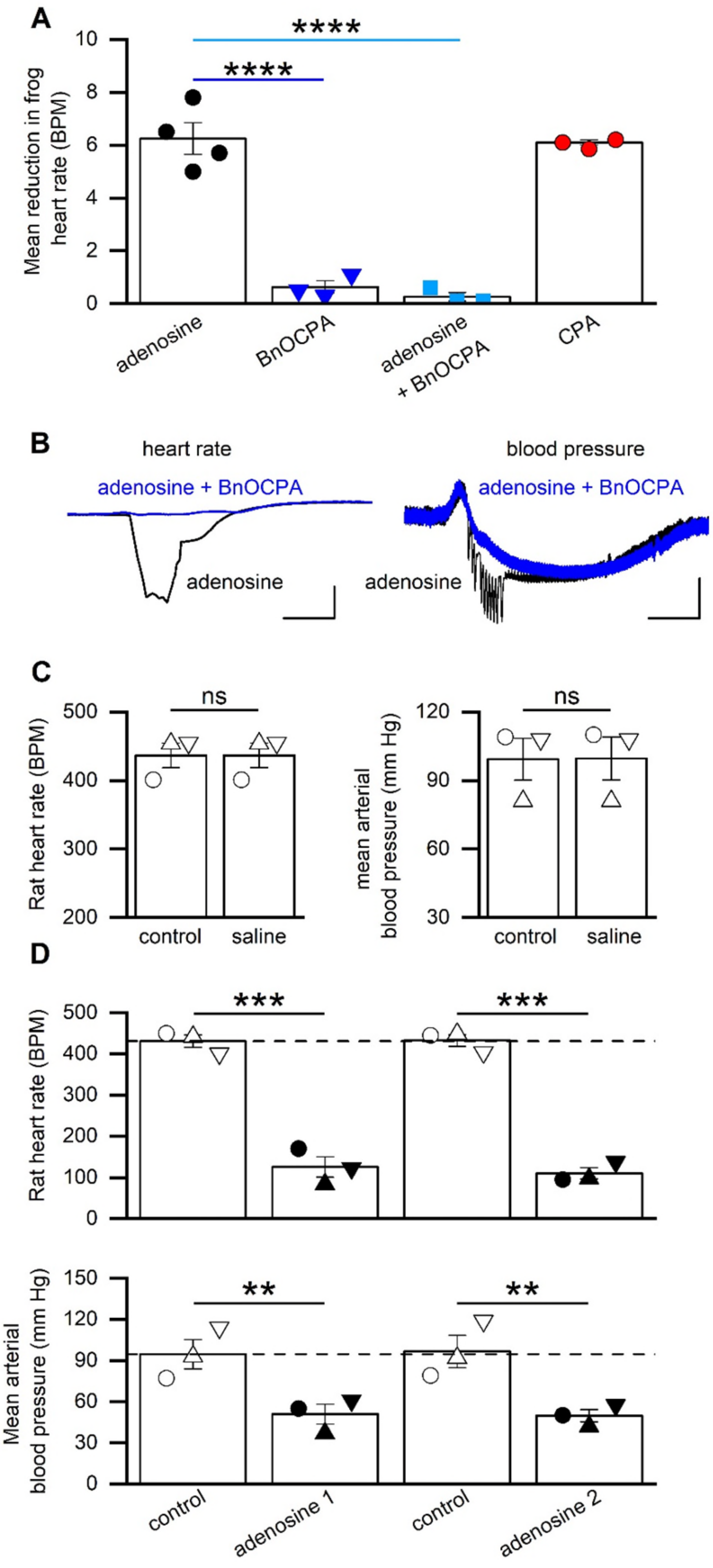


**Supplementary Fig. 10. Actions of BnOCPA on frog heart rate and controls for anaesthetised rat experiments.**

**Supplementary Fig. 10. Actions of BnOCPA on frog heart rate and controls for anaesthetised rat experiments.**

**A**, Data summary for 3 - 4 isolated frog heart preparations. Application of adenosine (30 μM) reduced heart rate (HR) from 41.8 ± 1.3 BPM to 35.5 ± 1.3 BPM. BnOCPA (300 nM) had no effect on HR (42.8 ± 1.2 BPM vs 42.1 ± 1.2 BPM; change 0.6 ± 0.2 BPM), an effect that was significantly different from that of adenosine (blue line; $P = 2.22 \times 10^{-5}$). BnOCPA significantly (cyan line; $P = 1.31 \times 10^{-5}$) reduced the effects of subsequent adenosine applications (from a reduction of 6.3 ± 0.6 BPM to 0.3 ± 0.2 BPM). CPA (300 nM) reduced HR by 6.1 ± 0.1 BPM, a value similar to that of adenosine. One way ANOVA on the difference in HR across the 4 conditions ($F(3,9) = 64.64$; $P = 2.070 \times 10^{-6}$), with the reported Bonferroni-corrected P values. Individual data points are presented, with the bar chart representing mean values ± SEM. **B**, Representative traces from a urethane-anaesthetised, spontaneously breathing rat. BnOCPA blocks the effect of adenosine on heart rate (left traces), but only prevents the early phase of adenosine-induced hypotension (right trace). Data taken from the trace in Fig. 5. Scale bars measure 100 BPM or 20 mm Hg and 6 s. **C**, Data summary for 3 urethane-anaesthetised, spontaneously breathing rats. Bar charts showing that injection of 0.9 % saline (equivalent volume to drug experiments) had no effect (two-tailed paired t-test) on either HR ($P = 1$) or mean arterial blood pressure (MAP; $P = 0.422$). **D**, Data summary for 3 urethane-anaesthetised, spontaneously breathing rats. Repeated adenosine injections have the same significant effect on HR ($P = 1.40 \times 10^{-4}$ and $1.02 \times 10^{-4}$, respectively) and MAP (P = 0.012 and 0.008, respectively) and thus show no run down. One-way RM ANOVA for both HR (Greenhouse-Geisser corrected $F(1.97,3.94) = 96.79$, $P = 4.48 \times 10^{-4}$, and MAP ($F(1.10,2.20) = 19.46$, $P = 0.040$) from 3 animals. In **C** and **D**, each symbol represents data from a single rat with the bar chart representing mean values ± SEM. ns, not significant; ∗∗, P < 0.02; ∗∗∗, P < 0.001; ∗∗∗∗, P < 0.0001.

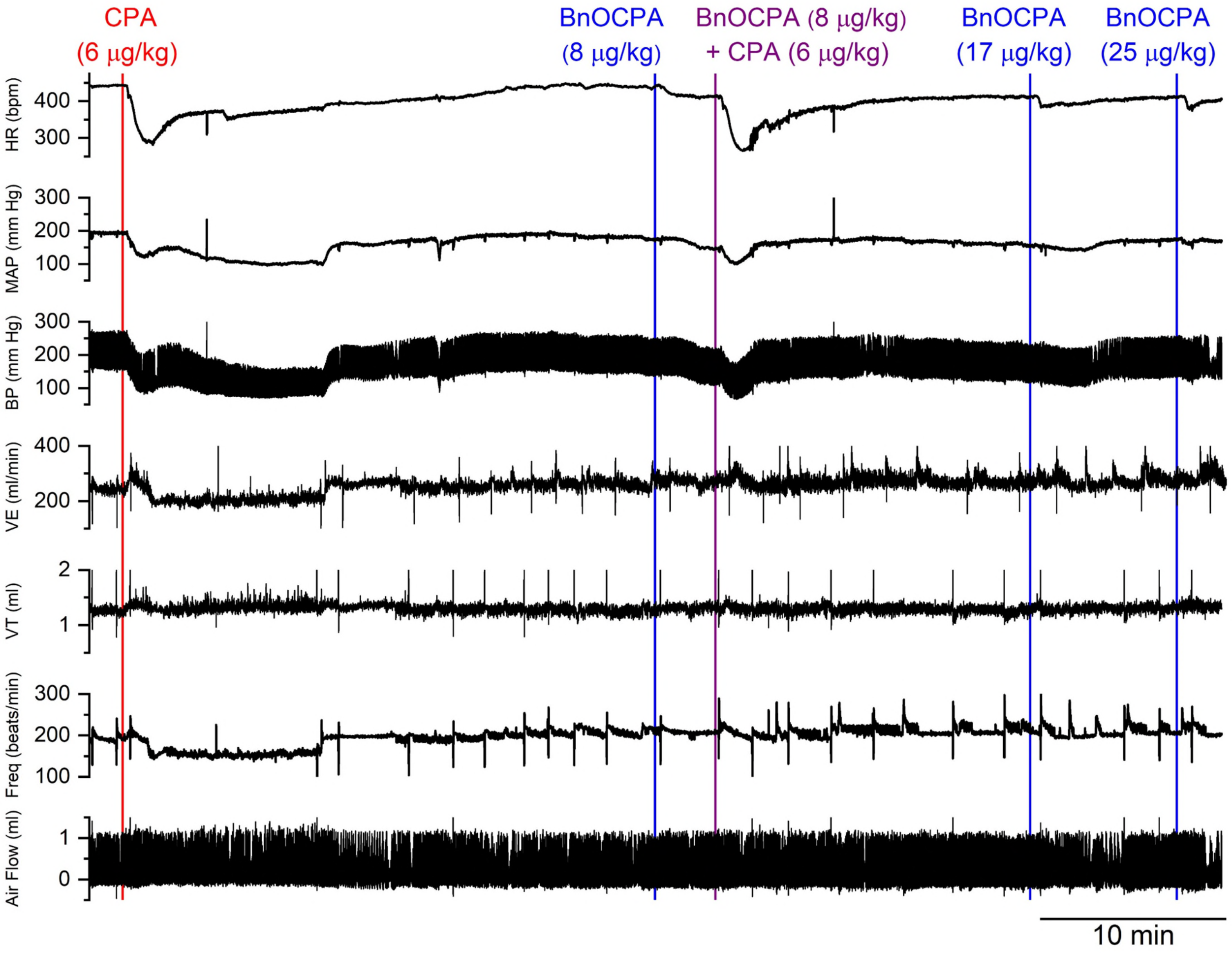


**Supplementary Fig. 11. BnOCPA has no actions on cardiorespiratory parameters, but antagonizes the effects of CPA.**

Examples of traces from a single spontaneously breathing urethane-anaesthetised rat showing: blood pressure (BP), from which heart rate (HR), and mean arterial pressure (MAP) are calculated, and tracheal tube airflow, from which respiratory frequency (Freq), tidal volume ($V_T$) and minute ventilation ($V_E$) are calculated. Applications of CPA (6 µg/kg; red vertical line), BnOCPA (8 µg/kg, 17 µg/kg, and 25 µg/kg; blue vertical lines), and the co-application (purple vertical line) of BnOCPA (8 µg/kg) and CPA (6 µg/kg) are shown by the vertical lines. BnOCPA and CPA were given as a 350 µL/kg IV bolus. The intravenous cannula was flushed with 0.9% saline to remove compounds in the tubing between drug applications. The second phase of the blood pressure response following the first dose of CPA is likely the result of the hyponea.

**Supplementary References 1 - 3**

1. Cordeaux Y, Ijzerman AP, Hill SJ. Coupling of the human A1 adenosine receptor to different heterotrimeric G proteins: evidence for agonist-specific G protein activation. *Br J Pharmacol* **143**, 705-714 (2004).
2. Baker JG, Hill SJ. A comparison of the antagonist affinities for the Gi- and Gs-coupled states of the human adenosine A1 receptor. *J Pharmacol Exp Ther* **320**, 218-228 (2007).
3. Hill SJ, Baker JG. The ups and downs of Gs- to Gi-protein switching. *Br J Pharmacol* **138**, 1188-1189 (2003).
