# Supplementary material for: Selective activation of Gαob by an adenosine A1 receptor agonist elicits analgesia without cardiorespiratory depression
Source: Nat Commun. 2022 Jul 18;13:4150. doi: 10.1038/s41467-022-31652-2 (PMC9293909; doi:10.1038/s41467-022-31652-2)
Supplement: Supplementary file 2 — Description of Additional Supplementary Files [file 41467_2022_31652_MOESM2_ESM.pdf]

Description of Additional Supplementary files

File name: Supplementary Movie 1
Description: Molecular dynamics dynamic docking simulation of BnOCPA binding to the apo A_1R

File name: Supplementary Movie 2
Description: Molecular dynamics dynamic docking simulation of HOCPA binding to the apo A_1R

File name: Supplementary Movie 3
Description: Molecular dynamics dynamic docking simulation of the Gob GαCT to the BnOCPA:A_1R complex.

File name: Supplementary Movie 4
Description: Molecular dynamics simulation of the BnOCPA:A_1R:Goa(α subunit) complex.

File name: Supplementary Movie 5
Description: Molecular dynamics simulation of the BnOCPA:A_1R:Gob(α subunit) complex.

File name: Supplemental Data 1
Description: BnOCPA pharmacokinetics Excel spreadsheet

File name: Supplemental Data 2
Description: Sum D Scripts (zipped folder)
