# Supplementary material for: Selective activation of Gαob by an adenosine A1 receptor agonist elicits analgesia without cardiorespiratory depression
Source: Nat Commun. 2022 Jul 18;13:4150. doi: 10.1038/s41467-022-31652-2 (PMC9293909; doi:10.1038/s41467-022-31652-2)
Supplement: Supplementary file 10 — Reporting Summary [file 41467_2022_31652_MOESM10_ESM.pdf]

nature portfolio

Corresponding author(s): Mark Wall, Graham Ladds, Bruno G. Frenguelli

Last updated by author(s): Jun 20, 2022

Reporting Summary

Nature Portfolio wishes to improve the reproducibility of the work that we publish. This form provides structure for consistency and transparency in reporting. For further information on Nature Portfolio policies, see our Editorial Policies and the Editorial Policy Checklist.

Statistics

For all statistical analyses, confirm that the following items are present in the figure legend, table legend, main text, or Methods section.

n/a	Confirmed	
☐	☒	The exact sample size (*n*) for each experimental group/condition, given as a discrete number and unit of measurement
☐	☒	A statement on whether measurements were taken from distinct samples or whether the same sample was measured repeatedly
☐	☒	The statistical test(s) used AND whether they are one- or two-sided<br>*Only common tests should be described solely by name; describe more complex techniques in the Methods section.*
☒	☐	A description of all covariates tested
☐	☒	A description of any assumptions or corrections, such as tests of normality and adjustment for multiple comparisons
☐	☒	A full description of the statistical parameters including central tendency (e.g. means) or other basic estimates (e.g. regression coefficient) AND variation (e.g. standard deviation) or associated estimates of uncertainty (e.g. confidence intervals)
☐	☒	For null hypothesis testing, the test statistic (e.g. *F*, *t*, *r*) with confidence intervals, effect sizes, degrees of freedom and *P* value noted<br>*Give P values as exact values whenever suitable.*
☒	☐	For Bayesian analysis, information on the choice of priors and Markov chain Monte Carlo settings
☒	☐	For hierarchical and complex designs, identification of the appropriate level for tests and full reporting of outcomes
☒	☐	Estimates of effect sizes (e.g. Cohen's *d*, Pearson's *r*), indicating how they were calculated

Our web collection on statistics for biologists contains articles on many of the points above.

Software and code

Policy information about availability of computer code

Data collection	Spike 2 version 7.08a (Cambridge Electronic Design, Cambridge UK), PClamp 10 (Clampex version 10.7.03 and Clampfit version 10.7.03 (Molecular Devices, San Jose CA USA), WinLTP version 2.01 (https://www.winltp.com), LabChart version 7.3.7 (AD instruments, Oxford UK), Modeller 9.19, pdb2pqr, propka3, HTMD1.13, VMD 1.9.3, ACEMD2, PLUMED 2.3. For cAMP and BRET assays, data was collected using MikroWin 2000 Software (Mithras LB 940 platereader) and exported as Excel files. Radioligand binding was measured on a Beckman Coulter LS 6500 Multi-purpose scintillation counter and data exported as Excel files.
Data analysis	OriginPro 2018-2021b, Prism 8.4, VMD 1.9.3, PLUMED 2.3, GetContacts. Bias plots and error calculations were performed using Excel for Mac v16.48. Customs scripts have been provided as a supplementary file.

For manuscripts utilizing custom algorithms or software that are central to the research but not yet described in published literature, software must be made available to editors and reviewers. We strongly encourage code deposition in a community repository (e.g. GitHub). See the Nature Portfolio guidelines for submitting code & software for further information.

Data

Policy information about availability of data

All manuscripts must include a data availability statement. This statement should provide the following information, where applicable:

- Accession codes, unique identifiers, or web links for publicly available datasets
- A description of any restrictions on data availability
- For clinical datasets or third party data, please ensure that the statement adheres to our policy

Data Availability: Source data are provided with this paper as a supplementary file. Customs scripts have been provided as a supplementary file. The active cryo-EM structure of the A1R (PDB code 6D9H) was retrieved from https://www.rcsb.org/structure/6d9h; the human neurotensin receptor 1 (PDB code 6OSA) from https://

www.rcsb.org/structure/6OSA, and the beta2 adrenergic receptor (PDB code 6E67) from https://www.rcsb.org/3d-view/6e67.

Field-specific reporting

Please select the one below that is the best fit for your research. If you are not sure, read the appropriate sections before making your selection.

- [x] Life sciences
- [ ] Behavioural & social sciences
- [ ] Ecological, evolutionary & environmental sciences

For a reference copy of the document with all sections, see nature.com/documents/nr-reporting-summary-flat.pdf

Life sciences study design

All studies must disclose on these points even when the disclosure is negative.

Sample size	Sample sizes adhered to the guidelines from the British Journal of Pharmacology (instructions to authors and the published guidelines editorial (Curtis M et al Br J Pharmacol. 2018 Apr;175(7):987-993).
Data exclusions	Data exclusion only occurred if there was technical failures or control experiments failed in which case the entire experiment was removed. All data analysis adhered to the guidelines described in - Curtis M et al Br J Pharmacol. 2018 Apr;175(7):987-993.
Replication	Experiments occurred over a period of time with various types of manipulations interleaved to avoid batch effects. Key observations replicated with different batches of animals and reagents and by different experimenters at least 2 - 3 times. Results across different labs working in different experimental systems were consistent with main theme and conclusions of the study
Randomization	For the pain studies, all animals were pre-screened for hypersensitivity (von Frey hair) when they arrived, and those that showed this i.e. a paw withdrawal threshold less than 8 were eliminated from the study. Those rats successfully developing mechanical allodynia (with PWT lower than 4g) were randomly assigned into different groups according to their PWT values of Pre-surgery, 1 week post-surgery and Pre-dosing to ensure a balanced distribution across groups. This is standard practice even in clinical trials. For the rotarod studies, rats were randomly assigned to treatment groups by animal care technicians not involved in the experimental studies. No randomization occurred for the in vitro studies where cells or slices from a common batch or animal, respectively, were exposed to a range of interleaved treatments. Similarly, no randomization occurred for the cardiovascular/respiratory studies studies in rats and frogs where the animals were exposed to the same protocol involving multiple drug treatments and where each animal served as its own control.
Blinding	In the pain and rotarod studies the experimenters were blinded to the reagents used. Blinding did not occur in the in vitro studies or those using frog heart or in the assessment of cardiorespiratory parameters. This is largely due to the exploratory nature of some of the studies (eg cardiac studies), the wide range of compounds and their concentrations, which would have made consistent blinding difficult to manage for the duration of the study, and the necessary use of positive controls in real-time assays, which compromises blinding.

Reporting for specific materials, systems and methods

We require information from authors about some types of materials, experimental systems and methods used in many studies. Here, indicate whether each material, system or method listed is relevant to your study. If you are not sure if a list item applies to your research, read the appropriate section before selecting a response.

Materials & experimental systems

n/a	Involved in the study	
x		Antibodies
	x	Eukaryotic cell lines
x		Palaeontology and archaeology
	x	Animals and other organisms
x		Human research participants
x		Clinical data
x		Dual use research of concern

Methods

n/a	Involved in the study	
x		ChIP-seq
x		Flow cytometry
x		MRI-based neuroimaging

Eukaryotic cell lines

Policy information about cell lines

Cell line source(s)	Cell line source: CHO-A1R cell lines were generated in Knight et al., 2016. The cells were provided by Dr Jurgen Muller, University of Warwick. The original source of these cells was from ATCC (https://www.atcc.org/products/ccl-61). Flp-In-CHO were from Thermo Fisher Scientific. HEK-293 were obtained from ATCC.
Authentication	Authentication. HEK293T cells from ATCC were validated as described in https://atcc.org/products/crl-3216. Authentication was by morphology as cells were grown.<br>Flp-In-CHO cells were validated as described in https://www.thermofisher.com/order.catalog/product/R75807. Authentication was by morphology as cells were grown.<br>CHO-K1-A1R cell lines were not authenticated in this study.
Mycoplasma contamination	All cell lines that enter the lab are firstly tested for microplasma contamination. All passed.
Commonly misidentified lines (See ICLAC register)	None

Animals and other organisms

Policy information about studies involving animals; ARRIVE guidelines recommended for reporting animal research

Laboratory animals	Sprague Dawley rats, males, postnatal days 12-20<br>Xenopus leavis frogs (young adult males ~9 months)<br>Adult male Sprague Dawley rats (230-330 g)
Wild animals	None
Field-collected samples	None
Ethics oversight	All experiments involving animals were conducted with the knowledge and approval of the University of Warwick Animal Welfare and Ethical Review Board, and in accordance with the U.K. Animals (Scientific Procedures) Act (1986) and the EU Directive 2010/63/EU. In vivo cardiorespiratory studies were conducted under the auspices of UK PPL 70/8936 and the chronic neuropathic pain studies under the auspices of P9D9428A9. Rotarod studies were approved by the Monash University Animal Ethics Committee in accordance with the Australian Code for the Care and Use of Animals for Scientific Purposes (2013) under Monash AEC protocol number 13333

Note that full information on the approval of the study protocol must also be provided in the manuscript.
